# Transfer Learning for Indoor Localization Algorithm Based on Deep Domain Adaptation

**DOI:** 10.3390/s23239334

**Published:** 2023-11-22

**Authors:** Jiahao Wang, Yifu Fu, Hainan Feng, Junxiang Wang

**Affiliations:** 1 School of Information and Software Engineering, University of Electronic Science and Technology of China, Chengdu 610000, China; wangjh@uestc.edu.cn (J.W.); fyf_uestc@163.com (Y.F.); hainan.feng@std.uestc.edu.cn (H.F.); 2 Technology Innovation Department of Tianfu Co-Innovation Center, University of Electronic Science and Technology of China, Chengdu 610000, China

**Keywords:** indoor localization, transfer learning, deep domain adaptation network

## Abstract

In application, training data and test data collected via indoor positioning algorithms usually do not come from the same ideal conditions. Changes in various environmental conditions and signal drift can cause different probability distributions between the data sets. Existing positioning algorithms cannot guarantee stable accuracy when facing these issues, resulting in dramatic reduction and the infeasibility of the positioning accuracy of indoor location algorithms. Considering these restrictions, domain adaptation technology in transfer learning has proven to be a promising solution in past research in terms of solving the inconsistent probability distribution problems. However, most localization algorithms based on transfer learning do not perform well because they only learn a shallow representation feature, which can only slightly reduce the domain discrepancy. Based on the deep network and its strong feature extraction ability, it can learn more transferable features for domain adaptation and achieve better domain adaptation effects. A Deep Joint Mean Distribution Adaptation Network (DJMDAN) is proposed to align the global domain and relevant subdomain distributions of activations in multiple domain-specific layers across domains to achieve domain adaptation. The test results demonstrate that the performance of the proposed method outperforms the comparison algorithm in indoor positioning applications.

## 1. Introduction

With the dawn of the era of digital intelligence, the importance of accurately acquiring location information between devices and users to optimize device–person interaction has become paramount. Consequently, indoor localization has risen to be one of the most critical technological components, finding extensive applications across various areas such as smart energy management, smart home systems, and points of interest identification [1,2,3]. It is often necessary to utilize alternative signal sources for indoor localization, such as Bluetooth Low Energy (BLE) [4], Ultra-wide Band (UWB) [5], Zigbee [6], Radio Frequency Identification (RFID) [7], Wi-Fi [8], etc. The majority of the above require the additional arrangement of specialized devices within the localization space, leading to issues such as high deployment costs and lack of convenience. BLE can turn all mobile devices carrying Bluetooth modules into signal transceiver devices, so its cost is very low and has higher accuracy than Wi-Fi. However, due to the poor stability of Bluetooth itself and its vulnerability to environmental interference, Bluetooth is more suitable as an auxiliary signal source to participate in the positioning process. Therefore, Wi-Fi signals have gained the favor of numerous researchers due to their ease of acquisition and widespread presence in various types of indoor scenarios.

Many localization methods based on Wi-Fi signals use Received Signal Strength Indication at the link layer as a data source. RSSI can only describe the overall superimposed result of the distorted signals received from different paths. Channel State Information can distinguish the specific conditions of signals propagating through different paths, so it is more stable than the RSSI on a timescale but has strong specificity over space [9]. Although CSI can provide fine-grained information, it will vary with the passage of time or changes in the environment, such as humidity, temperature, and furnishings [10]. It will cause domain shifts and different probability distributions between the training data collected during the offline stage and the test data obtained during the online stage. Directly using these data for training and locating will significantly reduce accuracy or even result in the failure of implementations. Reconstructing the model periodically in response to environmental changes can be a solution to mitigate the interference of domain shift, but the resampling process is costly and infeasible in real applications.

To solve the domain shift problem, transfer learning has been enrolled in many solutions and proven to provide strong robustness and high accuracy. But in real applications, this method still does not perform well because they only learn shallow representation features to find a transformation form in a low-dimensional manifold to make the subspaces of the source and target domains similar after mapping, which can only slightly reduce the domain discrepancy [11,12,13,14,15]. Recent research has shown that through the strong feature extraction ability of deep neural networks, more transferable features for domain adaptation can be learned from raw data. Therefore, applying deep neural networks to domain adaptation methods can achieve better domain adaptation effects.

In this work, we propose a Deep Joint Mean Distribution Adaptation Network (DJMDAN) to align the global domain and relevant subdomain distributions of activations in multiple domain-specific layers across domains for domain adaptation. Our work can roughly achieve the same distribution after domain adaptation and retain the fine-grained information between different categories at the same time. The experiment results show that the proposed DJMDAN method can effectively mitigate the interference in the data distribution caused by domain shift and improve localization accuracy in dynamic environments.

The main contributions of this work can be summarized as follows. First, we present an improved domain adaptation method, which performs global and local adaptation of the data domain at the same time under the premise of avoiding the loss of fine-grained information of the data as much as possible. Second, the deep TL method is introduced into indoor localization to reduce the overhead caused by resampling fingerprint points and solve the problem caused by the instability of CSI data due to time and environmental changes. Third, we implement a localization system based on the proposed method and prove the accuracy and robustness in complex and changeable indoor environments by evaluating the performance of this system in a real environment.

The rest of this paper is structured as follows. Section 2 discusses the relevant work of the proposed method. Section 3 provides a general overview of DJMDAN architecture and introduces its domain adaptation loss function components. In Section 4, we evaluated the proposed system on data collected from real-world scenarios and compared it with other transfer learning methods. Finally, Section 5 concludes the paper and discusses future work.

## 2. Related Work

Localization in dynamic environments can be considered a domain adaptation problem. When conducting positioning, usually, all features of the entire sample will be input into the model. This can cause feature values sensitive to environmental changes to vary significantly in distribution, thus rendering the model trained in the source domain inapplicable in the target domain. Therefore, to solve this problem, some researchers have proposed methods based on feature selection. The main idea of this kind of method is to reduce the impact of environmental changes on positioning by selecting features with strong robustness (i.e., insensitive to environmental changes). In the early stage of positioning transfer learning, the team of Sinno Jialin Pan [16] proposed to learn a low-dimensional latent feature space through dimensionality reduction so that the distribution of source domain data and target domain data in this space is approximate. Appropriate samples are selected from each fingerprint point for localization [17]. The method developed in [18] introduces a new relevance measure to select channels to be used as link features, thereby reducing the localization classification error when using the random forest classifier. On the other hand, after the device changes, the different number of access points will also seriously affect the localization accuracy. The main idea behind pseudo-labeling is to identify correlations between the source and target domain distributions, using source domain labels to annotate target domain data with pseudo labels. To solve the positioning offset problem after floor changes, the authors generate pseudo labels for the unlabeled target domain data, facilitating the mapping between domains [19]. In order to eliminate the impact of environmental changes on the localization results, Sun Zhuo et al. [20] established a dimensionality reduction method, which learns a mapping between a source data set and a target data set in a low-dimensional space.

To overcome the loss caused by shallow feature adaptation, researchers tried to apply deep learning in transfer learning and utilize the strong feature extraction ability of deep networks to achieve more transferable local features from data to optimize domain adaptation performance [21], and they hope to achieve better knowledge transfer results compared with those shallow domain adaptation algorithms. Most of the transfer learning methods based on deep domain adaptation can be mainly divided into two branches: the statistic moment matching-based method and the adversarial loss-based method.

The method of deep domain adaptation mainly depends on matching and minimizing the difference between two domains by aligning the first-order or second-order moment of domain features. Therefore, the network can learn the domain-invariant features. In this process, solutions relying on characteristics of Maximum Mean discrepancy (MMD) [22,23,24,25,26,27], Central Moment Discrepancy (CMD) [28], or covariance in a second-order statistic [29] have been widely researched. Another meaningful adversarial loss-based deep domain adaptation method makes use of data from different domains and keeps data labels non-discriminatory [30,31,32,33]. It makes use of the generative adversarial network (GAN) and tries to align the source domain and target domain at the data distribution level under the condition of unsupervised target domain.

In fact, most deep transfer learning methods are used in the field of image, and there are few related studies applied in the field of indoor localization. The deep transfer learning method is introduced into indoor localization [34], and the authors show through experiments that for a trained localization model, its feature extraction layer can be directly transferred to other models. It only needs the fully connected layer to be retrained to achieve the same baseline accuracy as that of the basic model without transfer. AdapLoc [35] aligns semantically with the source domain by marking a small number of CSI fingerprints as their positions in the target domain. This approach alleviates the alignment confusion issue to some extent. However, it does not align both inter-domain and intra-domain at the same time and uses Euclidean distance as the measure of domain features, which cannot adequately reflect the difference in the probability distribution of deep features.

Most of the existing deep domain adaptation algorithms only consider the global domain adaptation to solve the domain shift issue, that is, aligning the global data without considering the relationship between the two subdomains. Although this method mitigates the interference of domain shift on data distribution to a certain extent, the mapped data are often prone to confusion, making the data unable to be accurately classified. FitLoc [36] completes cross-room transfer by combining FLDA with Bregman divergence. It takes into account the fine-grained alignment problem, but the transfer ability is relatively weak.

To solve this problem, the proposed DJMDAN can adapt to the overall distribution difference between the two domains’ data and retain the fine-grained information between different categories of data at the same time. It effectively solves the confused alignment issue while ensuring the domain adaptation effect to achieve greater environmental adaptability and higher accuracy of indoor localization.

## 3. DJMDAN Algorithm

### 3.1. Model Architecture

**Figure 1:**
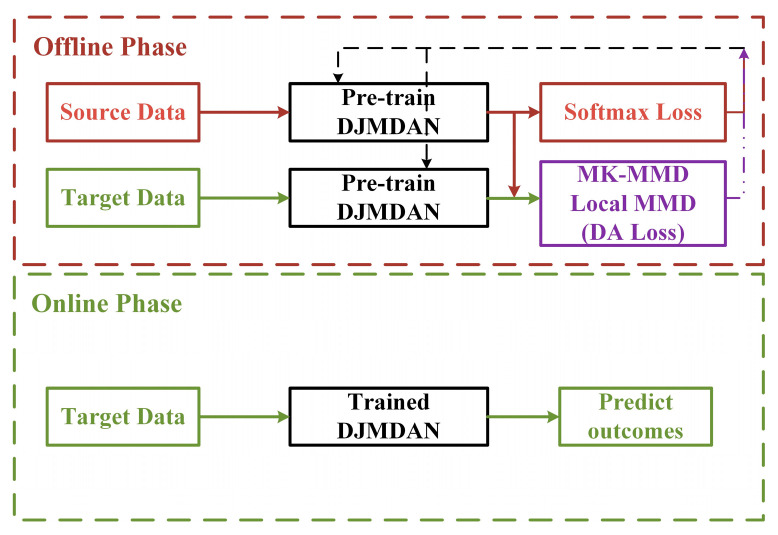
Flowchart of the DJMDAN algorithm.

**Figure 2:**
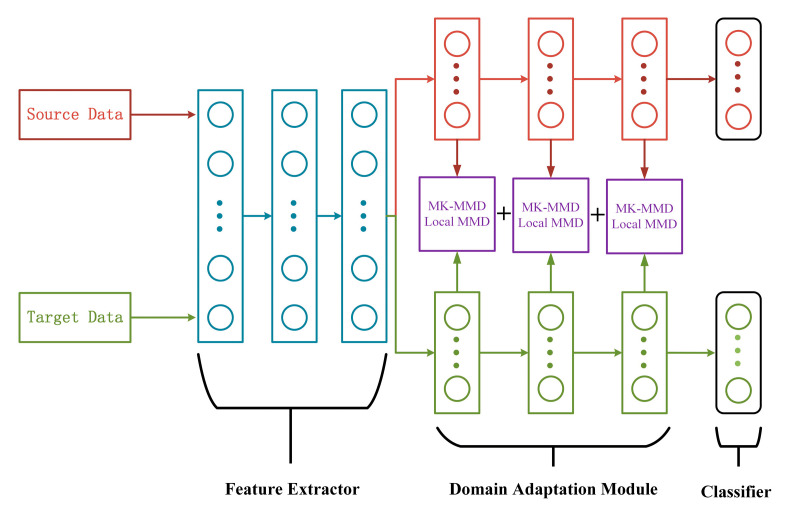
Diagram of the DJMDAN network structure.

The DJMDAN uses a deep neural network to improve domain adaptation performance by learning and updating the network by comparing the Multi-Kernel Maximum Mean Discrepancy (MK-MMD) [37] and Local Maximum Mean Discrepancy(LMMD) [38] of two domains output by the fully connected layer, and at the same time takes into account the classification loss of the source domain. The main process is shown in Figure 1. The proposed DJMDAN is based on a one-dimensional convolutional neural network, and the whole network is composed of three parts: feature extractor, domain adaptation module and classifier. The specific network architecture is shown in Figure 2.

As listed in Table 1, the feature extractor uses a three-layer composite layer, including convolution and pooling. It is used to extract more transferable features from the original data. The domain adaptation module consists of three fully connected layers. There is a domain adaptation layer to calculate the network’s feature transfer capability after each fully connected layer, and the last fully connected layer is used for the classification layer.

$D_{src}$ represents the source domain data collected in the offline phase, while $D_{tar}$ represents the unlabeled data collected in the online phase, and $y_{src}$ is the corresponding label. With the classification error and the domain adaptation error combined, the final loss function of DJMDAN is shown in (1), where $l_{clf}$ is the classification loss, and $l_{m}$, $l_{c}$ are the domain adaptation loss at the marginal and conditional probability distributions, respectively. α and β are the weighting factors that balance the two parts.

(1) $$l_{total}=l_{clf}\left(D_{src},y_{src}\right)+\alpha l_{m}\left(D_{src},D_{tar}\right)+\beta l_{c}\left(D_{src},D_{tar}\right)$$

### 3.2. Domain Adaptation Loss Function

Most of the existing domain adaptation solutions in deep transfer learning localization focus on adapting to the distribution differences of both domains from a global perspective. Although these methods effectively mitigate domain shift affection, the local geometric characteristics of the data are also ignored. They do not consider the relationship between the same category of data in the two domains, resulting in the loss of fine-grained information in the mapped data. It leads to the confused alignment problem of two domains, finally affecting the accuracy of the locating algorithm. To solve this problem, DJMDAN takes the MK-MMD and LMMD to achieve the adaptation to both global probability distribution and local probability distribution of two domains on the premise of avoiding fine-grained information loss as far as possible.

#### 3.2.1. Marginal Probability Distribution Adaptation

The MMD metric was often used as a measurement method in previous deep transfer learning methods based on statistical moment matching. However, MMD only uses a single kernel function to calculate the distribution difference between the two domains after mapping, so it cannot dynamically obtain the optimal kernel function according to the difference of the output data when calculating the distribution difference of the multi-layer network output data. As a result, the accuracy and adaptability of previous depth transfer methods are limited. To this end, MK-MMD is introduced as a measure of the difference between marginal probability distributions. MK-MMD originated from Multi-Kernel Learning (MKL) [39], which is a linear combination of multiple MMD based on different kernel functions. The composite kernel function composed of multiple basis kernel functions has multifarious advantages of different kernel functions. Therefore, compared with MMD with a single fixed kernel, MK-MMD has a stronger feature mapping ability, so it can help the network achieve better domain adaptation. The specific calculation formula of MK-MMD is shown in (2).

(2) $$\begin{array}{l} l_{m}\left(D_{src},D_{tar}\right)={\left\|\frac{1}{n_{s}}\sum_{i=1}^{n_{s}}\phi \left(x_{src}\right)-\frac{1}{n_{t}}\sum_{j=1}^{\;n_{t}}\phi \left(x_{tar}\right)\right\|}_{H}^{2}\\ =\frac{1}{n_{s}{}^{2}}\sum_{i=1}^{n_{s}}\sum_{j=1}^{n_{s}}\left(x_{src},x_{src}\right)-\frac{2}{n_{s}n_{t}}\sum_{i=1}^{n_{s}}\sum_{j=1}^{n_{t}}\left(x_{src},x_{tar}\right)\\ +\frac{1}{n_{t}{}^{2}}\sum_{i=1}^{n_{t}}\sum_{j=1}^{n_{t}}\left(x_{tar},x_{tar}\right) \end{array}$$

Since the kernel function of MK-MMD is composed of multiple different basis kernel functions, its kernel function κ (∙) can be specifically expressed as (3)

(3) $$K≜\left\{\kappa \left(\cdot \right)=\sum_{i=1}^{M}\mu_{i}k_{i}:\sum_{i=1}^{M}\mu_{i}=1,\mu_{i}\geq 0,\forall i\right\}$$

where M represents the number of basis kernel functions $k_{i}$ that form the MK-MMD kernel function, and $\mu_{i}$ represents the weight value corresponding to the basis kernel function $k_{i}$. Common basis kernel functions are listed in Table 2. 

Domain adaptation modules are considered to be added after each of the multiple fully connected layers to calculate the difference in the marginal probability distribution of the output, which can better reduce the difference in marginal distribution.

We assume that the output of two domains after the $K_{th}$ layer, respectively, is ${\left\{m_{a}^{sk}\right\}}_{a=1}^{n_{s}}$ and ${\left\{m_{b}^{tk}\right\}}_{b=1}^{n_{t}}$. The loss function for the entire difference in the marginal probability distribution can be expressed as (4).

(4) $$\begin{array}{l} l_{m}=\sum_{l=k1}^{k2}\left\|\frac{1}{n_{s}}\sum_{a=1}^{n_{s}}\phi \left(m_{a}^{sk}\right)-\frac{1}{n_{t}}\sum_{b=1}^{\;n_{t}}\phi \left(m_{b}^{tk}\right)\right\|\\ =\frac{1}{n_{s}{}^{2}}\sum_{l=k1}^{k2}\left[\sum_{a=1}^{n_{s}}\sum_{b=1}^{n_{s}}\kappa \left(m_{a}^{sk},m_{b}^{sk}\right)\right]\\ -\frac{2}{n_{s}n_{t}}\sum_{l=k1}^{k2}\left[\sum_{a=1}^{n_{s}}\sum_{b=1}^{n_{t}}\kappa \left(m_{a}^{sk},m_{b}^{tk}\right)\right]\\ +\frac{1}{n_{t}{}^{2}}\sum_{l=k1}^{k2}\left[\sum_{a=1}^{n_{t}}\sum_{b=1}^{n_{t}}\kappa \left(m_{a}^{tk},m_{b}^{tk}\right)\right] \end{array}$$

#### 3.2.2. Conditional Probability Distribution Adaptation

The above adaptation method based on marginal probability distribution only solves the problem of the overall distribution alignment of the two domains from the perspective of global changes and ignores the fine-grained information between data categories in the mapping process, which leads to the loss of information in the mapped data. To further improve the final output accuracy, the difference in conditional probability distribution between the two domains still needs to be adapted by aligning the distribution of the subset data belonging to the same category in both domains. Therefore, we used LMMD rather than MMD as the measurement method to calculate the distribution difference in the subset data of different categories, respectively. The weighted sum of them is the final conditional distribution probability difference, as is shown in (5).

(5) $$LMMD_{H}\left(p,q\right)=\frac{1}{C}\sum_{c=1}^{C}{\left\|\sum_{i=1}^{n_{s}}\omega_{i}^{sc}\phi \left(x_{i}\right)-\sum_{j=1}^{\;n_{t}}\omega_{j}^{tc}\phi \left(x_{tar}\right)\right\|}_{H}^{2}$$

In the above equation, $w_{i}^{sc}$ and $w_{j}^{tc}$ represent the weight of data belonging to certain category C, and $\sum_{i=1}^{n_{s}}w_{i}^{sc}=\sum_{j=1}^{n_{t}}w_{j}^{tc}=1$. Taking the source domain data $x_{k}$ as an example, the corresponding weight $w_{k}^{c}$ is calculated as follows.

(6) $$\omega_{k}^{c}=\frac{y_{kc}}{\sum_{\left(x_{i},y_{i}\right)\in D_{src}}y_{ic}}$$

where $y_{kc}$ is the $c_{th}$ element in the corresponding label vector of $x_{k}$. The original labels can be transformed into a one-hot vector to calculate the corresponding weights $w_{i}^{sc}$ because there are labels in the source domain. However, there are no labels of the target domain in the indoor localization field, so the weights $w_{j}^{tc}$ for the target domain data $D_{tar}$ without labels can be obtained via the soft labels output by the network.

(7) $$\begin{array}{l} l_{c}=\sum_{l=k1}^{k2}\frac{1}{C}\sum_{c=1}^{C}{\left\|\sum_{a=1}^{n_{s}}\omega_{a}^{sc}\phi \left(m_{a}^{sk}\right)-\sum_{b=1}^{\;n_{t}}\omega_{b}^{tc}\phi \left(m_{b}^{tk}\right)\right\|}_{H}^{2}\\ =\sum_{l=k1}^{k2}\frac{1}{C}\sum_{c=1}^{C}\left[\sum_{a=1}^{n_{s}}\sum_{b=1}^{n_{s}}\omega_{a}^{sc}\omega_{b}^{sc}k\left(m_{a}^{sk},m_{b}^{sk}\right)\right]\\ +\sum_{l=k1}^{k2}\frac{1}{C}\sum_{c=1}^{C}\left[\sum_{a=1}^{n_{t}}\sum_{b=1}^{n_{t}}\omega_{a}^{tc}\omega_{b}^{tc}k\left(m_{a}^{tk},m_{b}^{tk}\right)\right]\\ -2\sum_{l=k1}^{k2}\frac{1}{C}\sum_{c=1}^{C}\left[\sum_{a=1}^{n_{s}}\sum_{b=1}^{n_{t}}\omega_{a}^{sc}\omega_{b}^{tc}k\left(m_{a}^{sk},m_{b}^{tk}\right)\right] \end{array}$$

To better mitigate the difference in the conditional distribution, it is also necessary to calculate the conditional probability distribution difference in the multi-layer domain adaptation layer. Assuming that the outputs of two domains in the $k_{th}$ layer of the network are ${\left\{m_{a}^{sk}\right\}}_{a=1}^{n_{s}}$ and ${\left\{m_{b}^{sk}\right\}}_{b=1}^{n_{t}}$, to better compute the mapping function $\phi \left(\cdot \right)$, the loss function $l_{conditional}\left(D_{src},D_{tar}\right)$ for the entire difference in conditional probability distribution can be expressed as (7).

## 4. Experiments

To verify the localization effect of the DJMDAN algorithm in a dynamic environment, we conducted comparative experiments with existing localization algorithms based on transfer learning and traditional machine learning algorithms in real scenarios.

### 4.1. Experimental Environments

**Figure 3:**
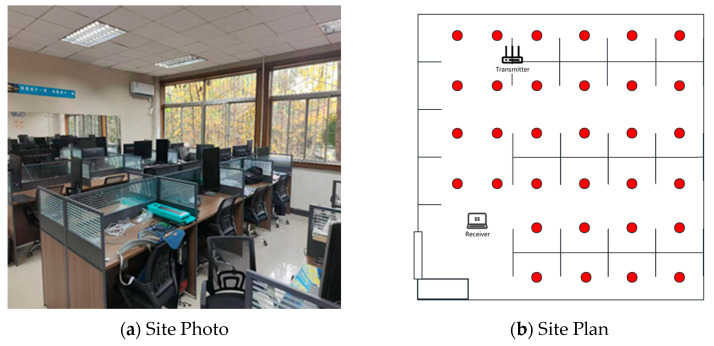
Environment setup.

The length and width of the experimental area is about 7 × 7 M^2^, and the actual photo and plan graph are shown in Figure 3.

A commercial router, TP-Link WDR7660, with three transmit antennas, was selected as the signal transmission device. The signal-receiving device was a Lenovo ThinkPad X201i laptop computer with Ubuntu 12.0.4 system and an Intel 5300 network card. The whole localization scene was divided into 32 grid-fingerprint points with 1 M spacing. The placements of devices and fingerprint points are shown in Figure 3b.

### 4.2. Evaluation Metrics and Comparison Algorithms

#### 4.2.1. Evaluation Criteria

Two criteria are mainly considered here to better evaluate the performance of localization algorithms.

**Average Error Distance (AED):** The arithmetic mean of the random error of all measurements taken in the experiments; the calculation formula is as follows.

(8) $$AED=\frac{1}{K}\sum_{i=1}^{K}\sqrt{{\left(x_{it}-x_{ip}\right)}^{2}+{\left(y_{it}-y_{ip}\right)}^{2}}$$

where $\left(x_{it},y_{it}\right)$ is the real coordinate value of the point to be measured, $\left(x_{ip},y_{ip}\right)$ is the predicted coordinate value, and K is the total number of samples in the test set.

**Cumulative Distribution Functions (CDF):** The probability distribution of the random variable X. In indoor localization, it is often used to reflect the proportion of the sample number in the overall sample number when the localization error value is less than a certain standard, and its expression is as follows:

(9) $$F_{D}\left(d\right)=P\left(D\leq d\right)$$

#### 4.2.2. Comparison Algorithms and Parameters

To evaluate the DJDMAN algorithm, we compare several algorithms mainly used in this field, such as KNN, TCA, 1D-CNN, DAN and DeepCORAL (which only uses the covariance of the fully connected layer output two domain data for the domain adaptation depth transfer method) [29].

Among them, 1DCNN is a deep learning algorithm without a domain adaptation module. Since the deep transfer learning method does not have a fixed basic network structure, the 1DCNN with three convolution-pooling layers and three fully connected layers is used as the basic network. 1DCNN is added as a benchmark algorithm to explore the influence of the domain adaptation method on the indoor localization effect. DAN is a depth transfer method that only uses the marginal probability distribution difference of data output by a fully connected layer for calculation, while DeepCORAL only uses the covariance.

For traditional machine learning methods, such as KNN and TCA, the K is set to 1. For deep learning methods, all 1D convolutional neural networks are used as their basic networks, and their parameters are shown in Table 1. In addition, the number of training rounds in the deep methods was set to 100, the learning rate was set to 0.001, the batch size was set to 128, and Adam was selected as the optimizer. To better suppress the noise activation problem in the early stage of training, the weight functions of two probability distributions were updated dynamically and gradually [40] in the experiment; therefore, $\alpha =\beta =2/1+e^{-K\delta }$, where K is a fixed constant equal to 10, and $\delta \in \left[0,1\right]$ increases linearly with training progress.

The DJMDAN uses four basic kernels, which are the linear kernel, polynomial kernel, Gaussian kernel and Laplacian kernel. As pointed out in [41,42], the weights of kernels in MK-MMD are set the same for better results, so it is not necessary to learn different weights. Therefore, we set the weights in multi-kernel MMD $\mu_{i}=1/4$ in this paper.

### 4.3. Results and Analysis

#### 4.3.1. Results under Varying Temporal Conditions

**Figure 4:**
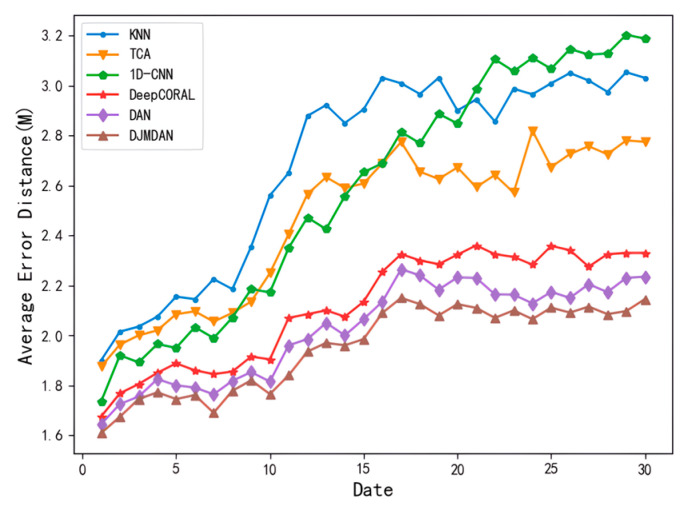
Comparison of the average positioning error of each positioning algorithm within 30 days.

To verify the effectiveness of DJMDAN with the change of time conditions, we collected CSI data within 30 days in the experimental environment for comparison. The data on the first day were collected as the fingerprint database data, that is, the source domain data in transfer learning. The data collected in the remaining days were used as the test data to represent the target domain data in transfer learning. Figure 4 shows the change in the average error distance for each localization algorithm over 30 days.

From the results, due to the strong feature extraction ability of deep networks, all deep learning methods maintain lower localization error in the early stage of the experiment. However, with the passage of time conditions, the domain shift level of CSI signals collected at different times gradually increases, and the localization error of the algorithm with a one-dimensional convolutional neural network begins to be gradually higher than that of other transfer learning methods and even worse than that of traditional machine learning methods. Although the localization error of the algorithm based on deep transfer learning increases under the condition of time change, its localization accuracy is still better than algorithms based on traditional transfer learning methods.

All deep learning methods use the same basic network, so they are consistent in terms of model scale. Among them, the proposed DJMDAN achieves a more accurate localization effect than two classical deep domain adaptation algorithms in the experiment. This is because DAN only considers the marginal probability distribution difference of two domains as the loss function when performing deep domain adaptation, which ignores the important factor of the conditional probability distribution difference. However, DeepCORAL only uses the covariance of two domains as the loss function, and the domain adaptation ability is also limited.

The DJMDAN performs domain adaptation operation on the multi-layer fully connected layer from the perspective of the joint probability distribution difference, which is more effective in mitigating the domain shift phenomenon of the two domains. As a result, better localization results are achieved.

**Figure 5:**
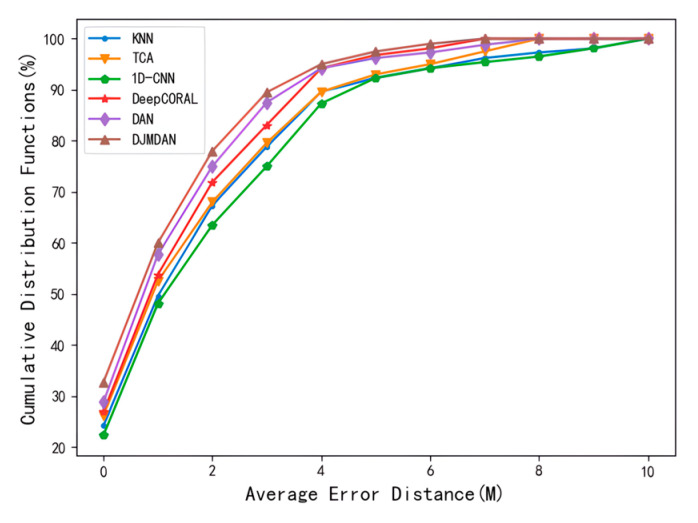
Comparison plot of the cumulative distribution function of error for each localization algorithm on day 22nd.

The cumulative distribution function diagram can better show the overall distribution of localization errors. As shown in Figure 5, the localization error of each algorithm on the 22nd day with larger discrimination and longer period among the localization errors of the above algorithms were selected to analyze its cumulative distribution. The result illustrates the CDF plot of the localization error for various transfer learning algorithms. The sample probability of localization results shows that the proposed DJMDAN algorithm is better than other algorithms in the same accuracy range under changing time conditions.

#### 4.3.2. Results under Varying Environmental Conditions

To examine the effectiveness of DJMDAN under other environmental conditions, we conducted experiments on three cases, which are door and window switches in the laboratory, the position of indoor lockers, and the condition changes of both changes at the same time.

**Figure 6:**
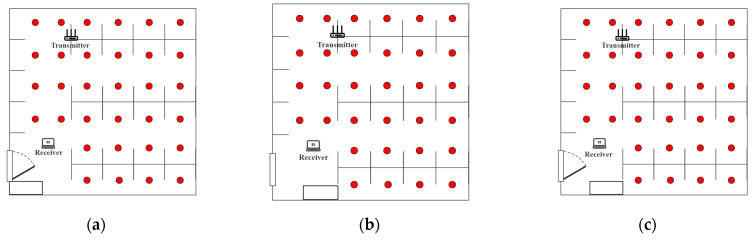
Changes in environmental conditions. (**a**) Laboratory Door and Window Configuration. (**b**) Laboratory Locker Placement Adjustment. (**c**) Combined Changes in Laboratory Door, Window, and Locker Placement.

**Figure 7:**
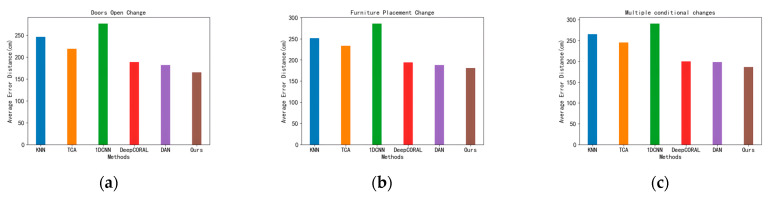
Results of each localization algorithm under the three environmental conditions. (**a**) Doors Open Change. (**b**) Furniture Placement Change. (**c**) Multiple conditional changes.

As shown in Figure 6, Figure 6a shows the opening of the door and window of the laboratory, Figure 6b shows the change in the placement of the lockers in the laboratory, and Figure 6c shows the change of both. Firstly, the CSI signal and location information of the fingerprint points were collected to form a fingerprint database without any indoor environmental conditions changes, and then the CSI signals of several points to be measured were collected as the location data for testing after each environmental condition was changed. Figure 7 shows the error of each algorithm under three conditions. Our solution shows the smallest average error distances in all tests.

**Figure 8:**
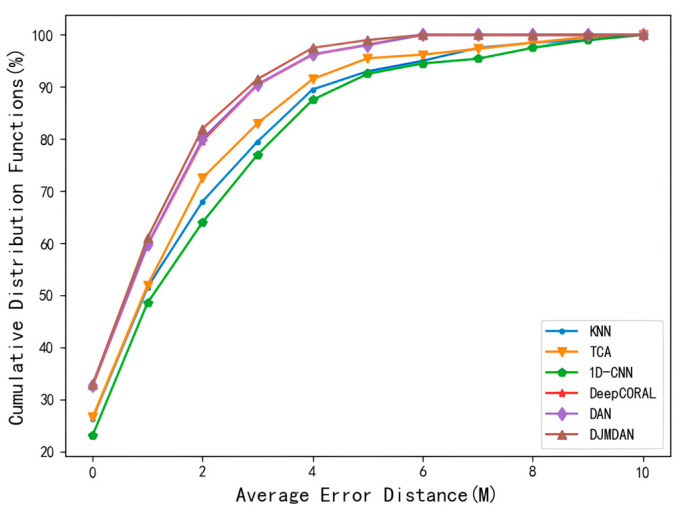
Results of cumulative distribution function for each localization algorithm under superimposed change in environmental conditions.

The test error in Figure 7c corresponding to the superimposed change in the environmental conditions was taken for further comparative analysis, and the error cumulative distribution function of each algorithm is shown in Figure 8. The results show that under the change of other environmental conditions, the sample probability of localization results of the proposed DJMDAN algorithm is better than other algorithms within the same limited accuracy range.

According to the experimental results, it can be seen that the proposed DJNDAN can obtain a better domain adaptation effect than the traditional transfer learning methods and achieves greater indoor locating accuracy because of the abstract features extracted using a deep one-dimensional convolutional neural network. In addition, DJMDAN considers the overall domain adaptation and the local perspective of the data simultaneously so that the fine-grained information of each category is retained in the domain adaptation process. Therefore, DJMDAN can maintain better robustness and obtain higher localization accuracy to resist environmental changes.

## 5. Conclusions

In this paper, a deep domain adaptation localization approach for Wi-Fi CSI signals using transfer learning has been proposed. It can maintain data domain adaptation, both global and local, by adapting the marginal and conditional probability distribution of two-domain data. The proposed method is an update based on DAN, and experiments have shown that our method outperforms models of the same scale that do not use transfer learning. It also surpasses DAN and other transfer learning methods based on MMD metrics and methods using CORAL metrics. Experimental results show that it can effectively solve the domain shift problem and maintain a relatively ideal localization accuracy in complex and changing indoor environments.

Moving forward, there are two potential areas for further exploration and improvement. Firstly, the current domain adaptation operation is still based on the overall perspective of data, and we could consider introducing some metric functions based on the internal geometric characteristics of the data for domain adaptation work. Secondly, we are currently using a relatively basic one-dimensional convolutional neural network structure. It would be beneficial to select deeper network models that are more suitable for CSI signals as the basic model for deep domain adaptation localization algorithms.

## Figures and Tables

**Table 1:** Network structure parameters.

Layer	Configuration
Conv1	(5,8), Stride = 2, Padding = 0
Maxp1	5, Stride 1
Conv2	(5,16), Stride = 2, Padding = 0
Maxp2	5, Stride 1
Conv3	(5,32), Stride = 1, Padding = 0
Max3	5, Stride = 1
FC1	512
FC2	256
FC3	128

**Table 2:** Common Basis Kernel Functions.

Kernel Name	Expression	Parameters
Linear kernel	$K\left(x_{i},x_{j}\right)={x_{i}}^{T}\cdot x_{j}$	
Radial basis kernel	$K\left(x_{i},x_{j}\right)=e^{-\gamma {\left\|x_{i}-x_{j}\right\|}^{2}}$	$\gamma =\frac{1}{2\sigma^{2}},\sigma >0$
Polynomial kernel	$K\left(x_{i},x_{j}\right)={\left(\gamma {x_{i}}^{T}x_{j}+k\right)}^{n}$	γ>0,k>0

## Data Availability

Data are contained within the article.
